# Epidemiology of newcastle disease in village chicken in melokoza district, gofa zone, southwest Ethiopia

**DOI:** 10.1016/j.heliyon.2023.e14384

**Published:** 2023-03-09

**Authors:** Yohannis Betela, Gizachew Hailegebreal, Dessie Shiferaw

**Affiliations:** a Melo Koza District Livestock and Fishery Development Office, Ethiopia; b Faculty of Veterinary Medicine, Hawassa University, P.O.Box, 05, Hawassa, Ethiopia

**Keywords:** Ethiopia, Melokoza, Newcastle disease, Poultry, Sero-prevalence

## Abstract

Poultry production contributes significantly to the livelihoods of Ethiopian farmers and the national economy, although it is hampered by different factors, including infectious diseases. A cross-sectional study was conducted from December 2019 to May 2020 in Melokoza District, Gofa Zone, Ethiopia. The objectives of the study were to estimate the seroprevalence of Newcastle disease in unvaccinated chickens and to identify the risk factors associated with the disease. A systematic random sampling technique was employed to select chickens from individual chicken owners. A total of 405 blood samples were collected and submitted to the national veterinary institute in Bishoftu, Ethiopia. Hemaglutination inhibition test was performed to detect antibodies from the collected chickens’ serum. Both univariable and multivariable logistic regression analyses were performed by using the STATA statistical software package. The test result showed that the overall seroprevalence was 68.8% (95% CI: 64%, 73%). The highest seroprevalence of 86% (58/67) was recorded in Gazar kebele (lowland), whereas the lowest seroprevalence of 45% (32/71) was recorded in Maizelo (highland). Sex, age, altitude and management practice risk factors showed significant associations (p < 0.05) with the disease prevalence. In conclusion, this study emphasized the prevailing higher prevalence of Newcastle disease in free-scavenging chickens. Regular vaccination for Newcastle disease is therefore recommended. Further studies are warranted to better understand the circulating strain and its economic effect on backyard poultry production in the study area.

## 1 Introduction

Poultry production makes a great contribution to poverty reduction programs in developing countries like Ethiopia. It is an immediate cash source for poor rural communities, generates employment opportunities, improves family food security and empowers women [1]. The optimum operation of poultry production is, however, hindered by a number of factors, including infectious diseases, such as Newcastle disease, Infectious Bursal Disease, Mycoplasmosis, Pasteurellosis, and Salmonellosis, to mention a few [2]. The economic losses suffered include high mortality, morbidity, and reduced meat and egg production. Additionally, the cost of treating and managing flocks during the course of the disease amplifies the magnitude of losses [3]. Newcastle Disease has a global distribution and is considered as one of the most important factors which impair the backyard poultry production in Ethiopia [4]. Newcastle disease virus (NDV) is a member of the genus Avian orthoavulavirus 1 within the new subfamily Avulavirinae of the family Paramyxoviridae [5].

Serological and virological evidence has shown that NCD is one of the major problems in village chickens and it is known to be endemic in Ethiopia [2,[6], [7], [8], [9], [10]]. The disease has become endemic in the poultry population and recurs every year, inflicting heavy losses [11]. The highest rate of NCD outbreaks from March to May has been suggested to be associated with the high rate of chicken marketing for Easter [12]. The main movement of chicken marketing is from the periphery to the center (from rural to town), which favors the spread of diseases all over the country [13].

Melokoza District is one of remotely located districts in Gofa zone, South Ethiopia where backyard poultry production is common. The poultry sector in this area is, however, affected by several technical and non-technical factors. The existence of infectious diseases is the biggest problem, causing significant impacts on chicken production in the area. NCD represents the most severe poultry disease responsible for marked economic losses in Ethiopia, but there is no sufficient epidemiological information in the country, particularly in village chickens. In the current study area, no study has been carried out. Therefore, the aim of this study was to estimate the seroprevalence of NCD and to assess the associated risk factors in Melokoza District, Gofa zone.

## 2 Materials and methods

### 2.1 Description of study area

The study was conducted from December 2019 to May 2020 in the Melokoza District of Gofa Zone, South West Ethiopia. The district is located 605 km south-west of Addis Ababa, Ethiopia's capital city. The district lies at a latitude of 80 13′ 19″ North and a longitude of 370 37′ 40″ East. Geographically, the district is bounded by East Geze Gofa, in West Konta special District/Salamago, in North Dawuro, and in South Basketo special District. The predominant agricultural production system in the area is a crop-livestock mixed farming system. The chicken population of six study kebeles was estimated to be 8081 [25]..

**Fig. 1:**
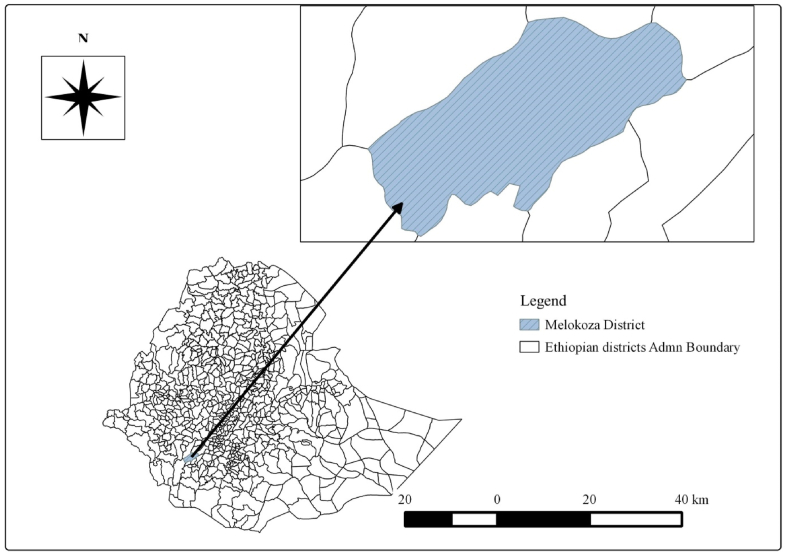
Map showing the study area (Melokoza district). The map depicted in Fig. 1 is our own developed from Ethiopian shape files using QGIS software.

### 2.2 Study design and sampling methods

A cross-sectional study design was carried out on free-scavenging chickens from December 2019 to May 2020 to estimate the seroprevalence of Newcastle disease virus antibodies and associated risk factors for the disease in Melokoza District.

A preliminary field survey was done to map out the distribution and concentration of local chickens. Six kebeles (the lowest administrative unit in Ethiopia) were purposively selected based on their poultry population and representation of three different altitude clusters (low, mid, and high altitudes) in the district. Because of the very challenging topography of the district, accessibility of the kebeles to the road was also considered to handle logistic issues while collecting samples and data. Accordingly, from each cluster, two kebeles were purposely selected; Gazar and Mender 4 from low altitude, Laha and Tata from mid-altitude, and Gaitsa and Maizelo from high altitude. The list of households that had three or more chickens was prepared with the help of community representatives, village leaders, and animal health technicians working in the kebeles. Households from each kebele and study subjects were selected by using a systematic random sampling technique. Farmers were informed and agreed to bring three chickens per household to the health post for blood sample collection in the early morning before the chickens went out for scavenging.

### 2.3 Study population and sample size determination

The study population comprised all apparently healthy chickens with a history of no vaccinations and was managed under a backyard production system. Most of the chickens (72%) were local breeds and the remaining (28%) were cross breeds (MKLFR, 2019). The sample size was calculated by using the formula described by Thrusfield [26]; by considering 50% expected prevalence (P), a 95% confidence interval and 5% desired absolute precision (d). So the calculated required sample size (N) was 384. A total of 405 samples were collected.

### 2.4 Study methods used

***Serum sample collection:*** Blood samples (2 ml) were collected using syringes after plucking a few feathers from the ventral surface of the humeral region of the wing and wiping the site with cotton damped with alcohol 70%. The syringes were labeled and placed horizontally over night at an angle of 45° to facilitate serum formation. After clotting of the blood, the syringes were returned to a vertical inverted position to separate the serum. The separated serum was transferred into cryovials with screw tops, labeled and stored at-20 °C in the Hawassa University veterinary laboratory until transported to the National Veterinary Institute (NVI), where the Haemagglutination inhibition test was performed.

***Haemaglutination-inhibition test (HAI):*** The HAI test was conducted according to the procedures recommended by OIE (2021). The test was undertaken at NVI, Bishoftu, Ethiopia, by running two fold dilutions of equal volumes (0.025 ml) of phosphate buffered saline (PBS) and test serum (0.025 ml) in U-bottomed micro titer plates. Four haemagglutinating units (HAU) of virus or antigen were added to each well, and the plate was left at room temperature for a minimum of 30 min. Finally, 0.025 ml of 1% (v/v) washed chicken red blood cells (RBCs) was added to each well and, after gentle mixing, the RBCs were allowed to settle for about 40 min at room temperature. The HAI titer was read from the highest dilution of serum, causing complete inhibition of 4 HAU of antigen. Only wells with RBCs that streamed at the same rate as the control wells (containing 0.025 ml of RBCs and 0.05 ml of PBS only) were considered positive for inhibition [4].

### 2.5 Operational definitions of variables

In this study, various risk factors that are associated with the occurrence of Newcastle disease were selected. Independent variables considered during this study were: sex, age, flock size, altitude, management, and awareness of NCD vaccination. Age groups: young (6 months) and adult (>6 months). Flock size: In this study, chicken numbers 1–10 per household are classified as a small flock, and anything above 11 is classified as a large flock. Poor management in this study means: no chicken house, no supplementary feed, and no water. Medium management: housed but with no supplementary feed and no water. Good management: housing, supplementary feed, and water. Low altitude is 1000 m above sea level, mid altitude is 1001–1700 m above sea level, and high altitude is > 1700 m above sea level.

### 2.6 Ethics approval and consent to participate

Ethical clearance for this study was obtained from University of Hawassa, College of Natural and Computational Sciences, Faculty of Veterinary Medicine, Minutes of Animal Research Ethics and Review committee (HUCNCS2019) [15]. Before conducting the research, participants were informed of the objectives of the study and written and signed consent was obtained from the chicken owners to be involved in the study and taking samples from their chicken.

### 2.7 Data management and analysis

Data collected from field and laboratory investigations were entered and coded in a Microsoft Excel spreadsheet (Microsoft Corporation) before being analyzed in Stata version 14 (StataCorp, 4905 Lakeway Drive, College Station, Texas 77,845, USA). The seroprevalence was calculated as the number of seropositive samples divided by the total number of samples tested. Logistic regressions reporting odds ratio and coefficient were performed to determine the association between various risk factors (explanatory variables) and the dependent variables (disease). Multicollinearity of risk factors was checked using Kruskal gamma statistics, and those non-collinear risk factors with p ≤ 0.25 in univariable logistic regression were subjected to multivariable logistic regression analysis. A p-value <0.05 was considered statistically significant.

## 3 Results

### 3.1 Seroprevalence of newcastle disease

A total of 405 serums were collected from free scavenging chickens and tested using the HAI test for the presence of NCD antibodies. The overall seroprevalence of NCD antibodies was 68.8%. The highest seroprevalence was recorded in Gazar, 58/67 (86.5%) and the lowest in Maizelo kebeles, 32/71 (45%) (Table 1).

**Table 1:** Seroprevalence of NCD in Melokoza District and selected kebele.

Sampled Kebeles	Number of samples	Negative	Positive	Prevalence (%)	95% CI
Gazar	67	9	58	86.5	85.3–98.6
Mender4	69	18	51	73.9	71.4–85.8
Tata	67	31	36	53.7	51.7–65.3
Laha	64	20	44	68.7	66.3–79.1
Gaitsa	67	10	57	85	84.6–97.2
Maizelo	71	39	32	45	43.6–57.2
Total	405	127	278	68.8	

CI: Confidence interval.

### 3.2 Analysis of risk factors for NCD occurrence in melokoza district

The association of risk factors considered in this study with the seroprevalence of NCD was analyzed by univariable logistic regression analysis. The analysis revealed that a significantly higher seroprevalence (p < 0.001) was recorded in female chickens (76%) than in males (33%). Also, there was a significant difference in the seroprevalence of NCD between the different age groups (p < 0.001), which was higher in young chickens (58%) than in adults (42%). Similarly, the seroprevalence of NCD antibodies was 48%, 27%, and 24% in poor, medium, and good management practices, respectively (Table 2). The difference in prevalence among various management practices was also statistically significant (p < 0.05).

**Table 2:** Univariable logistic analysis of risk factors in relation to NCD serostatus in free scavenging chickens in Melokoza District.

Variable	Category	No of chickens tested	Prevalence (%)	OR (95% CI)	P value
Sex	Female	272	67%	1.45 (1.24–1.98)	0.000
	Male	133	33%	–	
Age	Adult	171	42%	–	
	Young	234	58%	2.9 (2.56–3.53)	0.000
Flock size	≤10 chickens	85	21%	–	
	>10 chickens	320	79%	1.5 (1.11–2.7)	0.12
New chicken to Flock	Yes	368	91%	2.3 (1.93–5.87)	0.99
	No	32	7%	–	
Altitude	Low	139	34%	1.7 (1.2–2.1)	0.08
	Mid	133	32%	–	
	High	135	33%	1.6(1.1–1.8)	0.091
Management	Poor	198	48%	2.46 (1.26–3.78)	0.005
	Medium	109	27%	1.67 (1.36–2.25)	0.024
	Good	97	24%	–	
Vet service	No	43	10%	–	
	Yes	162	40%	1.75 (1.29–1.35)	0.35

OR፡ Odd ratio; CI፡ confidence interval.

The multivariable logistic regression analysis revealed that among the risk factors considered in the analysis (Table 3), sex, age, altitude, and management practices were associated with seropositivity (p < 0.05). The results showed that female chickens had 1.45 times higher odds of acquiring the disease than males. The odds of NCD antibody seroprevalence were observed to be significantly higher by 2.9 times in young chickens than in aged ones. Chickens reared in low altitudes had 1.7 times higher odds of being positive for NCD antibodies than those chickens reared in mid and high altitudes. Similarly, poorly managed chickens had higher odds of NCD antibody positivity than those managed in medium and good conditions (Table 3).

**Table 3:** Multivariable logistic regression analysis of risk factors association for seroprevalence of NCD in free scavenging chickens in Melokoza District.

Variable	Category	No of chickens tested	Prevalence (%)	OR (95% CI)	P value
Sex	Female	272	67%	1.45 (1.24–1.98)	0.000
	Male	133	33%	–	
Age	>6 month	171	42%	–	
	≤6 month	234	58%	2.9 (2.56–3.53)	0.000
Altitude	Low	139	34%	1.7 (1.2–2.1)	0.008
	Mid	133	32%	–	
	High	135	33%	1.4(1.1–2.1)	0.091
Management	Poor	198	48%	2.46 (1.26–3.78)	0.005
	Medium	109	27%	1.67 (1.36–2.25)	0.024
	Good	97	24%	–	

OR፡ Odd ratio; CI፡ confidence interval.

## 4 Discussion

The overall seroprevalence of NCD in scavenging chickens in Melokoza was 68.8%. This higher seroprevalence is an indication that NCD is a major health constraint for village chickens in the study area.

The current study's findings are lower than previous reports from different parts of the country [10,14,16]. However, it is higher than some other reports from different areas of Ethiopia, which range from 11.6 to 61.6 [6,9,10,[17], [18], [19], [20]]. The variability in prevalence report shown by this study and other reports from different regions could be due to differences in study parameters or exposure to mild viral strains that induced immunity but did not killed many chickens. The presence of lentogenic or possibly mesogenic NCDV in backyard poultry flocks in an area can result in a constant cycle of infection that periodically boosts the immunity of all exposed chicken, resulting in a higher proportion of chicken with antibodies [2]. Another reason for the differences between studies could be subjectivity and differences in the HAI cutoff values used to interpret the results. The difference in geographical location, high contact between free scavenging chickens, and dissimilarity in management practices could also have a role in the sero-status variation [21].

In the present study, the association between the sex of chickens with the seroprevalence of NCD was statistically significant (p = 0.000). Females were more likely to be infected with NCDs (67%) than males (33%). Female chickens were 1.45 times more affected than male chickens. This finding is in agreement with the reports of Tadesse et al. (2005) and Geresu et al. (2016) [10,19].

The age of the chickens was significantly associated (p = 0.000) with the seroprevalence of NCD. A higher seroprevalence of NCD was observed in younger chickens (58%) than in adults (42%). This finding is consistent with some studies in the country [18,22] and also in other areas [1,23]. The villages’ poultry populations are characterized by involvement of multiple factors, and the highest morbidities were reported in the young chickens. Young chickens without maternal antibodies are very susceptible to NCD and when infection occurs, it results in heavy losses [21].

There was a significant (p = 0.008) variation in the seroprevalence of NCD across the altitude in the study areas. The seroprevalence was higher in chickens reared in low altitudes than in mid-altitudes and high altitudes. The prevalence status of 34%, 32%, and 33% has been registered in low, mid, and high altitudes, respectively. This result is in general agreement with reports from various parts of Ethiopia [6,10,18,22]. The higher seroprevalence of NCD at low altitude might be related to the difference in chicken population, which was lower at higher altitude. Variations in prevalence across the three altitudes could also be attributed to ecological characteristics of a specific area, such as climate, settlement pattern, sanitary and socioeconomic practices, which may facilitate the spread of the disease [24].

## 5 Conclusions

The overall seroprevalence of 68.8% reported in unvaccinated chickens in this study shows that NCDV has been established and is circulating in the area. The prevailing NCD seroprevalence registered in the free scavenging chicken population indicates the importance of the disease in the study area. Sex, age, altitude variation, and various management practices were statistically identified as the major risk factors for NCD seropositivity. Therefore, more attention should be given to the study area and adjacent regions by adopting prophylaxis through the use of NCD vaccines for the chickens. More research is needed to fully understand the circulating strain and its economic impact on backyard poultry production in the study area.

## Ethics approval and consent to participate

Ethical clearance was obtained from Hawassa University College of Natural and Computational Sciences research and review committee.

## Declarations

### Author contribution statement

Gizachew Hailegebreal: Conceived and designed the experiments; Analyzed and interpreted the data; Wrote the paper.Yohannis Betela: Conceived and designed the experiments; Performed the experiments; Analyzed and interpreted the data; Contributed reagents, materials, analysis tools or data; Wrote the paper.Dessie Shiferaw: Analyzed and interpreted the data; Wrote the paper.

### Funding statement

This research did not receive any specific grant from funding agencies in the public, commercial, or not-for-profit sectors.

### Data availability statement

Data will be made available on request.

### Declaration of interest's statement

The authors declare no competing interests.

## References

[1] Mossie T. (2018). Newcastle disease in Ethiopia: a review on Epidemology, diagnosis, control and other methods. Birtish Journal of Poultry Science.

[2] Chaka H., Goutard F., Bisschop S.P.R., Thompson P.N. (2012). Seroprevalence of Newcastle disease and other infectious diseases in backyard chickens at markets in Eastern Shewa zone, Ethiopia. Poultry Sci..

[3] Corkery G., Ward S., Kenny C., Hemmingway P. (2013). Computer Aided Process Engineering-CAPE Forum 2013.

[4] OIE (2021).

[5] Rima B., Balkema-Buschmann A., Dundon W.G., Duprex P., Easton A., Fouchier R., Consortium I.R. (2019). ICTV virus taxonomy profile: Paramyxoviridae. J. Gen. Virol..

[6] Zeleke A., Sori T., Gelaye E., Ayelet G. (2005). Newcastle disease in village chickens in the southern and rift valley districts in Ethiopia. Int. J. Poultry Sci..

[7] Fentie T., Heidari A., Aiello R., Kassa T., Capua I., Cattoli G., Sahle M. (2014). Molecular characterization of Newcastle disease viruses isolated from rural chicken in northwest Ethiopia reveals the circulation of three distinct genotypes in the country. Trop. Anim. Health Prod..

[8] Mulisa D.D., Alemu R.B., Keno M.S., Furaso A., Heidari A., Chibsa T.R., Chunde H.C. (2014). Characterization of Newcastle Disease Virus and poultry-handling practices in live poultry markets, Ethiopia. Springer Plus.

[9] Chaka H., Goutard F., Gil P., Abolnik C., Servan De Almeida R., Bisschop S., Thompson P.N. (2013). Serological and molecular investigation of Newcastle disease in household chicken flocks and associated markets in Eastern Shewa zone, Ethiopia. Trop. Anim. Health Prod..

[10] Tadesse S., Ashenafi H., Aschalew Z. (2005). Seroprevalence study of Newcastle disease in local chickens in central Ethiopia. Int. J. Appl. Res. Vet. Med.

[11] Dessie T., Jobre Y. (2004). A review of the importance and control of Newcastle disease in Ethiopia. Ethiopian Veterinary Journal.

[12] Spradbrow P.B. (1999). March. Epidemiology of Newcastle disease and the economics of its control. Poultry as a Tool in Poverty Eradication and Promotion of Gender Equality–Proceedings of a Workshop.

[13] Dessie T., Ogle B. (2001). Village poultry production systems in the central highlands of Ethiopia. Trop. Anim. Health Prod..

[14] Birhan M., Birhan M., Tesfaye S., Tariku A. (2019). Detection of antibodies against Newcastle and infectious bursal disease on chicken in north Gondar zone, Ethiopia. Journal of Animal and Feed Research.

[15] Tadesse H., Belete S., Deress B. (2018). Evaluation of the safety and efficacy of combined. J. Vet. Med..

[16] Anebo Z.G., Teklemichael K., Bacha B., Habte T., Hunde A. (2014). Evaluation of the newcastle disease antibody level after vaccination regimes in chickens in Debrezeit Agricultural Research Center, Ethiopia. Journal of Veterinary Medicine and Animal Health.

[17] Endale M.A. (2017). Sero prevallence of Newcastle disease in scavenging chicken in selected district of aris zone. International journal of agriculture and Veterinary scince.

[18] Derbew G., Getachew B., Haftu B. (2016). Seroprevallence of Newcastle disease and its associated risk factors in village chicken. Int. J. Exp. Diabetes Res..

[19] Geresu M.A., Elemo K.K., Kassa G.M. (2016). Newcastle disease: seroprevalence and associated risk factors in backyard and small scale chicken producer farms in Agarfa and Sinana Districts of Bale Zone, Ethiopia. Journal of Veterinary Medicine and Animal Health.

[20] Terefe D., Belaineh R., Chaka H., Sombo M., Mekuria A., Gugsa K.L., Damena D. (2015). Serological and molecular study of Newcastle disease virus in village chickens in selected rift-Valley areas, Ethiopia. J. Vet. Sci. Technol..

[21] Awan M.A., Otte M.J., James A.D. (1994). The epidemiology of Newcastle disease in rural poultry: a review. Avian Pathol..

[22] Getachew B., Kyule M.N., Balcha M., Dawo F. (2014). Seroprevallence of newcastle disease virus antibodies in village chickens in kersana-kondality district, Ethiopia. Global Vet..

[23] Bouzari M., Mousavi M.R. (2006). Sero Epidemology of Newcastle disease in demostic village chickens of plain areas of Isfaham province. Iran. J. Vet. Res..

[24] McCallum H., Barlow N., Hone J. (2001). How should pathogen transmission be modelled?. Trends Ecol. Evol..

[25] MKLFR (2019).

[26] Thrusfield M. (2005).

