# Supplementary material for: Epidemiology of newcastle disease in village chicken in melokoza district, gofa zone, southwest Ethiopia
Source: Heliyon. 2023 Mar 9;9(3):e14384. doi: 10.1016/j.heliyon.2023.e14384 (PMC10023967; doi:10.1016/j.heliyon.2023.e14384)
Supplement: Multimedia component 1 [file mmc1.docx]

Questionnaire format for data collection

Dear participant,

This study is an investigation “*Epidemiology of Newcastle Disease in Village Chicken in Melokoza District, Gofa Zone, Southwest Ethiopia*”. The results of this study will help veterinarians and public institutions in designing control strategies.

Hence, your genuine opinion and response will help as to get detailed and reliable information to complete the study successfully. You will in no way be personally linked to any of the results of the survey. There is no risk to you from participating in this questionnaire, and there is no anticipated direct benefit. Thank you in advance for your participation!

1. **Demographic characteristics of the households/farms in the study area**
   1. Name of kebele where household/ farm located ______date______ Telephone_________
   2. Name of respondent/farm manager ___________________________ Sex ____Age_____
   3. Education status of the respondent /farm manager:

Primary school ______

Secondary school _______ college and university _______

- 1. How long have you been working with chicken keeping? _______
  2. Farm ownership: private___________ government_________

1. **Flock characteristics**
   1. Chicken number at present _____________

How many internal flocks you have? ________

- 1. Breed type of chicken: _______________
  2. Sources/foundation of poultry breeds:

Purchased from Government/Private Hatchery_____________

Provided by agricultural research center___________

If others (specify it) ______________

- 1. How many chickens did you buy to start with? _________
  2. What age of chicken did you buy? ____________
  3. Are there particular times of year when you will buy or not buy? __________
  4. What time(s) of year you have most chicken in the farm? ___________

1. **Housing condition**
   1. Chicken house management

Deep litter ________ Cage system_________

Housing condition, poor (allow rodents) ________ good (standardized) ________

- 1. Hygienic status of your farm, poor (not clean) _________ good (clean) _________
  2. How often is the litter cleaned? __________
  3. How is waste dealt with? (Burning, fertilizer etc.)________

1. **Feeding and watering practices**
   1. When do you feed your chickens?

Morning & evening __________

Morning & afternoon ___________

Morning, afternoon & evening _________

- 1. How frequent do you provide water?

Free access __________ morning only _________morning and evening only _________

- 1. What is the source of water?

Well water ________, river ________, tape water _________, pond water __________

1. **Health management and diseases**
   1. Interaction of chicken with other chicken or wild birds. Yes ________no_________
   2. Presence of other animal species living with chicken? Yes _______no_______
   3. Presence of rodents in your chicken resting area? Yes _______no________
   4. Separate house for sick chicken (yes__________, No__________)
   5. Disposal of dead chicken in the compound (yes__________, No__________)
   6. Disease outbreak in your farm? Yes ________no_________
   7. Major clinical sign of the disease that cause major losses?

Respiratory sign__________________________________________________________

Digestive sign____________________________________________________________

Nervous sign ____________________________________________________________

Mixed signs _____________________________________________________________

Death of birds____________________________________________________________

In which season does morbidity and mortality is high? Rainy season _____Dry season___

- 1. Which age group of chicken is mainly affected? young_____________ Adult_________

1. **Extension /animal health service**
   1. Do you have advisory service on poultry production? Yes_________ No________
   2. How do you get poultry health care services?

From employed animal health professional________________

From part-time animal health professional_________________

From private or public vet clinic_______________

- 1. Does your chicken get protective vaccination? Yes________ No___________

If yes, for which diseases? __________________________________________________

- 1. Do you provide chemoprophylaxis to your chickens? Yes_______ No________

If yes, which drugs used _______________________________?

- 1. Is there access to poultry vaccines or drugs in the market? Yes_______ No__________

1. **Poultry production and marketing**
   1. Do you have market access to buy poultry production inputs? Yes________ No________
   2. Do you have market access to sell your poultry products? Yes __________ No_________
2. **List major constraints you are facing to enhance poultry production in your farm**
   1. Presence of diseases_____________________________________
   2. Shortage of feed from surrounding_________________________
   3. Lack of market to sell products____________________________
   4. Lack of access to replacement stock________________________
   5. Lack of access to other poultry inputs_______________________
   6. Lack of time to do farm work activities_________________________
   7. Lack of adequate veterinary services in the near by_______________________
   8. Lack of knowledge about scientific poultry management practices__________________
   9. Other, if any_____________________________________
3. Knowledge of Newcastle disease YES/NO
4. Have you ever heard about Newcastle disease __________
5. Heard from whom? Animal health worker_____ veterinarian_________ community____
6. Clinical signs in chicken _______________________
7. Does it transmit from chicken to chicken _____________
8. Does it cause illness in human ______________________
9. Attitude about Newcastle disease virus YES/NO
10. Do you think Newcastle disease serious illness in chicken ___________
11. Do you think cleaning chicken house frequently is necessary? __________
12. Do you think you need more information on Newcastle? ___________
13. Can Newcastle disease be prevented in chicken? __________________
14. What are prevention means(vaccine, isolation, disposable of dead birds) ________________
15. Do you know is there any vaccine for NCDV? Yes/No
16. Are you involved in when the Newcastle vaccination campaign on to prevent the disease? Yes/No
